# Welcome Back: Responses of Female Bonobos (*Pan paniscus*) to Fusions

**DOI:** 10.1371/journal.pone.0127305

**Published:** 2015-05-21

**Authors:** Liza R. Moscovice, Tobias Deschner, Gottfried Hohmann

**Affiliations:** Department of Primatology, Max Planck Institute for Evolutionary Anthropology, Leipzig, Germany; University of Freiburg, GERMANY

## Abstract

In species with a high degree of fission-fusion social dynamics, fusions may trigger social conflict and thus provide an opportunity to identify sources of social tension and mechanisms related to its alleviation. We characterized behavioral and endocrine responses of captive female bonobos (*Pan paniscus*) to fusions within a zoo facility designed to simulate naturalistic fission-fusion social dynamics. We compared urinary cortisol levels and frequencies of aggression, grooming and socio-sexual interactions between female bonobos while in stable sub-groups and when one “joiner” was reunited with the “residents” of another sub-group. We hypothesized that fusions would trigger increases in aggression and cortisol levels among reunited joiners and resident females. We further predicted that females who face more uncertainty in their social interactions following fusions may use grooming and/or socio-sexual behavior to reduce social tension and aggression. The only aggression on reunion days occurred between reunited females, but frequencies of aggression remained low across non-reunion and reunion days, and there was no effect of fusions on cortisol levels. Fusions did not influence patterns of grooming, but there were increases in socio-sexual solicitations and socio-sexual interactions between joiners and resident females. Joiners who had been separated from residents for longer received the most solicitations, but were also more selective in their acceptance of solicitations and preferred to have socio-sexual interactions with higher-ranking residents. Our results suggest that socio-sexual interactions play a role in reintegrating female bonobos into social groups following fusions. In addition, females who receive a high number of solicitations are able to gain more control over their socio-sexual interactions and may use socio-sexual interactions for other purposes, such as to enhance their social standing.

## Introduction

In contrast with the majority of primate species, bonobos (*Pan paniscus*) live in patrilineal social groups characterized by male life-long residency and female dispersal at sexual maturity, typically into groups without other relatives present [1]. Other primate species with a bias towards male philopatry and female dispersal include the closely related chimpanzee (*Pan troglodytes sp*.*)*, spider monkeys (*Ateles sp*.) and ancestral and modern humans [2], [3]. *Pan* and *Ateles* social systems share further parallels with humans in their high degree of fission-fusion social dynamics, referring to the fluid splitting and merging of individuals within sub-groups of varying size, composition, and spatial cohesion [4]. Due to these features, female members of the *Pan* and *Ateles* genera arguably experience more variable social environments, requiring more frequent interactions with non-kin, than is typical of female primates. It is interesting then that social relationships among female bonobos have been characterized by high levels of gregariousness and cooperation and low levels of aggression, patterns more typically associated with the philopatric sex in the majority of primate species [5] including chimpanzees [6] and *Ateles* sp. [7]. In the wild, female bonobos are disproportionately represented in sub-groups in comparison with males [8] and share preferred, potentially monopolizeable foods primarily with other females [9]. While several studies indicate the potential for social conflict and aggression among female bonobos [10], [11], intra-sexual aggression often occurs too infrequently to identify linear dominance relationships among females (e.g. [12]-[14]). Given the evidence for high levels of social tolerance among female bonobos, and the parallels in social systems with humans, a better understanding of potential sources of social conflict among female bonobos and any strategies that females may use to avoid or reduce conflict has implications for understanding the evolution of human cooperation.

For species characterized by a high degree of fission-fusion social dynamics, fusions represent sources of potential social tension, since they may trigger uncertainty concerning social interactions, increased competition over access to resources, or conflicts of interest related to coordination of activities [4]. In several species with high rates of fission and fusion, including spider monkeys (*Ateles geoffroyi*) [15], chimpanzees [16] and hyenas (*Crocuta crocuta*) [17], rates of aggression increase following fusions and occur selectively between individuals who have been apart [15], [17]. While little is known about short-term physiological responses to fusions, instability in social relationships and unpredictable aggression more generally can be potent psychological stressors, triggering the release of cortisol from the adrenals in a range of primate species [18], including bonobos [19]. Circulating cortisol concentrations can be measured non-invasively in bodily fluids, providing a reliable indicator of perceived stress [20].

Investigations of fission-fusion social dynamics in bonobos are useful for characterizing the extent of social tension among females, and for exploring how females may reduce or resolve social conflicts. However research on responses of bonobos to fusions is lacking, likely due to difficulties in monitoring changes in group composition in the wild and observing post-fusion events that may involve simultaneous, widely-dispersed social interactions among group members. Several sanctuaries and zoos have facilities designed to incorporate naturalistic fission-fusion social dynamics into ape husbandry (e.g. [21]), providing opportunities to examine responses to fusions under more controlled settings. Due to spatial limitations in captive environments, simulated fissions are usually incomplete, in that separate sub-groups often maintain partial auditory, visual and/or olfactory contact. This differs from conditions in the wild, where sub-groups often remain separated over large distances following fissions. However, research in other species indicates robust behavioral responses to simulated fissions and fusions in captivity (spider monkeys: [22], chimpanzees: [23]), suggesting that such simulations may be biologically relevant for species with a high degree of fission-fusion social dynamics. While field studies are critical for understanding fission-fusion social dynamics, captive studies may also provide insights into relevant behavior and physiology surrounding fusion events.

We characterized behavioral and endocrine responses of captive female bonobos to fusions within subgroups of varied composition at the Frankfurt Zoo Ape Facility, which was designed to simulate natural fission-fusion social dynamics. In each fusion event, one female subject transferred from one sub-group to another, whose members she had been separated from for varying periods of time. Through this design we were able to examine individual responses to fluctuating social environments. We predicted that fusions may trigger increased social tension among female bonobos, which is likely to be expressed through increases in aggression and cortisol levels. Alternatively, in a species characterized by high levels of female gregariousness and cooperation, fluctuations in female sub-group membership may pose little threat to females. If this is the case, then rates of aggression and cortisol levels should remain similar to baseline conditions before fusions, and should not be influenced by changes in the social environment.

Female bonobos may also have behavioral strategies to reduce or avoid the escalation of social conflict following fusions. This hypothesis is consistent with evidence in other species with a high degree of fission-fusion social dynamics that fusions are associated with increases in ritualized social interactions, including embraces among spider monkeys [15] and chimpanzees [24] and pseudo-penile displays among female hyenas [17], that have been linked to reduced social tension in some species [15]. In bonobos, two likely candidate behaviors for reducing social tension are grooming and socio-sexual behavior, which among female bonobos consists primarily of genital contacts in which females embrace and move their hips laterally while keeping their vulvae in contact [25]. Grooming has an established role in maintaining social relationships and reducing social tension in primates [26]. While in many primate species grooming occurs primarily among close relatives or peers of similar age and rank [27], in bonobos grooming occurs frequently among unrelated females and preferences for grooming partners appear to fluctuate over time [1], suggesting more flexibility in grooming relationships than is typical in other primate species. While largely absent among other primate species, genital contacts are common among female bonobos, and appear to serve multiple functions including facilitating the integration of newly immigrant females into social groups [28], reducing social tension, especially in feeding contexts [29], [30], an expression of social status by high-ranking females [25] and a strategy for low-ranking females to increase their social standing by selectively advertising their socio-sexual interactions with higher-ranking partners [31]. If grooming and/or socio-sexual interactions help to reduce or avoid social tension and aggression following fusions, then we predict that: 1) One or both of these behaviors should increase among females who have been apart, 2) Females who receive grooming and/or socio-sexual solicitations from other females will be less likely to aggress against them and 3) Females who receive higher rates of post-fusion grooming or socio-sexual interactions will have reduced cortisol levels following fusions. Additionally, if grooming and/or socio-sexual interactions are important in tension reduction, then they should be initiated more often by females who experience more uncertainty in their social interactions following fusions. These may include: 1) A female who joins a sub-group that has not had other changes in composition for several days, as she must then re-assess social relationships with several individuals simultaneously, 2) Lower-ranking females who may be more likely targets of aggression or 3) Females who have been separated from each other for longer periods.

## Material and Methods

Data were collected from September through November 2011 at the Frankfurt Zoo, Germany. All behavioral data and urine samples were collected non-invasively, in accordance with NIH published standards and with the management practices of the Frankfurt Zoo Bogori Wald Ape Facility. The study protocol was approved by an ethics panel of the Frankfurt Zoo headed by Dr. Thomas Wilms, Head Mammal Curator (approval received on 7^th^, June 2011). The bonobo colony consisted of six mature females (five parous and one nulliparous), three mature males and five immatures. One female (MI) and one male had been transferred into the group one-month prior to the beginning of the study. Two of the mature females (KA and KU) were siblings, but they had been reared in different zoos without contact prior to adulthood. Females were at different reproductive stages during the study (Table 1), but all were sexually active and showed cyclic fluctuations in the appearance of their sexual swellings. Sexual swellings were scored daily on a four-point scale based on their firmness and skin surface structure [32]. Since there is evidence that receptivity and sexual activity may increase coinciding with the period of maximal tumescence [33], females were only focal subjects on days when their swellings were not maximally tumescent. We also tried to insure that non-focal females in each sub-group represented a range of different reproductive states. This reduced the possibility that variation in behavioral responses across different fusion events may be influenced by differences in reproductive state.

**Table 1:** Description of female subjects, the number of urine samples provided and average daily cortisol levels on non-reunion and reunion days.

*Subject*	*Dominance category*	*Age (yrs)*	*Reproductive state*	*# Observation days (Non-reunion/Reunion)*	*# Urine samples (Non-reunion/Reunion)*	*Mean (*± *SD) daily cortisol levels (ng/mg Cr)*, *Non-reunion/Reunion*
NT	High	48	Cycling / lactating	8 / 10	18 / 21	192.1 ± 23.5 / 172.6 ± 32.5
KA	High	25	Cycling / lactating	7 / 10	12 / 21	142.4 ± 17.9 / 149.6 ± 51.1
KU	Mid	13	Cycling / lactating	9 / 9	12 / 12	188.0 ± 69.9 / 108.6 ± 28.8
ZO	Mid	14	Pregnant	9 / 8	9 / 9	226.3 ± 14.1 / 265.4 ± 62.5
MA	Low	61	Cycling	6 / 12	6 / 6	142.1 ± 22.8 / 102.7 ± 64.2
MI	Low	10	Cycling	11 / 6	12 / 12	154.0 ± 14.2 / 190.9 ± 36.6

During the study period, the colony was housed in two adjacent indoor viewing exhibits (approx. 440 and 1100 cubic meters) and also had access to several off-exhibit enclosures (from 90–170 cubic meters). The off-exhibit enclosures had fencing on one side, facilitating urine sample collection for hormone analyses. The viewing exhibits and off-exhibit enclosures were connected via remotely controlled sliding gates. Viewing exhibits contained naturalistic climbing structures and were regularly enriched with novel objects and foraging tasks. As another form of environmental enrichment and for management purposes, the colony was sporadically separated into sub-groups in different viewing exhibits for varying periods of time before being reunited, in a process simulating the fission-fusion dynamics of wild bonobos. The colony was fed an assortment of fruits and vegetables twice daily, at 9:30 and 14:00, and also received supplemental snacks throughout the day. To insure that feeding interactions did not influence behavior, all observations occurred outside of feeding times.

After initially remaining together for 48 hours, colony members were separated into two sub-groups, each consisting of three adult females with their associated offspring and one to two adult males. Division of individuals into sub-groups typically occurred during feeding times, when individuals could choose to feed in either of the two indoor viewing exhibits. This approach was chosen to approximate fissions among wild bonobos, which often occur when some individuals initiate independent travel to forage in a different location. In order to insure that each sub-group included equal numbers of females representing a range of reproductive states, and also to insure that reunions involved as many different dyadic combinations as possible, bonobos were sometimes assigned to a specific sub-group, which was facilitated by limiting access to various enclosures other than the one containing the designated sub-group. While this additional strategy meant that individuals did not always freely choose their sub-group associations, this strategy was used infrequently during this study and was familiar to the bonobo colony, since the zoo keepers used the same strategy to move individuals between sub-groups and enclosures periodically for management purposes and for cage cleaning.

Each sub-group was housed in one of the indoor viewing exhibits and also had daily access to two off-exhibit enclosures. During separations, sub-groups had limited visual, auditory and olfactory, but no physical contact. We conducted one-hour continuous focal observations daily at 8:00, 10:30 and 12:00. On *non-reunion days*, a focal female was observed in her sub-group without changes in sub-group composition. On *reunion days*, which occurred within two days of non-reunion days, a focal female (*the joiner*) was reunited with the members of the other sub-group (*the residents*) at 8:00, after being separated from them for between 2–15 days (median = 7). In the wild, bonobos can remain in separate parties for hours to weeks following fissions [34].

As part of their daily husbandry, sub-groups spent between one to two hours each morning in their off-exhibit enclosures while the viewing exhibits were cleaned. As a result, across non-reunion and reunion days the first behavioral observation occurred in the off-exhibit enclosures, while the second and third observations occurred in the viewing exhibits. Reunions occurred in the off-exhibit enclosures, facilitated by a 30 cubic meter passageway connecting the enclosures of the two sub-groups. Reunions only occurred when a joiner voluntarily entered this passageway, from which she could enter the enclosure of the other sub-group. Following the last daily behavioral observation, the joiner was moved back to her original sub-group, restoring the non-reunion sub-group composition. After every female member of one sub-group had been individually reunited with the members of the other sub-group, two new sub-groups were formed using the same procedure described above and the process was repeated until each focal female had been observed on a minimum of two non-reunion and two reunion days.

During focal observations we recorded the initiation and direction of grooming and the frequency, direction and outcome of socio-sexual solicitations involving the focal female and any sub-group members. Intra-sexual solicitations were scored when one female faced another and squatted with open legs, presenting her genitals ventrally to the intended target. Solicitations sometimes involved additional behaviors including vocalizations, head movements or thrusting of hips, but these behaviors were rarely performed without ventral genital presentations, and this behavior was used as the decisive measure of a solicitation. Inter-sexual solicitations were identified by: 1) Dorsal or ventral presentation of genitals by females to a male target or 2) Penile displays directed at a female target. The outcome of each solicitation was recorded as: Accepted (solicitor and target approach, followed by either dorso-ventral mounting or ventro-ventral genital contact) or rejected (target ignores the solicitation and often moves away from the solicitor). Following a rejection, if individuals renewed their solicitations this was scored as a new solicitation event. Socio-sexual interactions that were not preceded by a clear solicitation (29.6%, N = 32) were excluded from analyses.

As a behavioral measure of social tension, we used all occurrence sampling [35] to record aggressive behaviors, defined as directed displays, charges or contact aggression and their outcomes (ignore, move away (at least 2 m), retreat (at least 5 m) or flee (rapid movement at least 5 m). Decisive aggressive interactions in which the target moved away, retreated or fled occurred too rarely to establish a linear dominance hierarchy. However, decisive aggressive outcomes were sufficient to classify females into rank categories, based on whether they typically won or lost any aggressive interactions that they were involved in. Females who won a higher proportion of aggressive interactions than they lost were categorized as high ranking. Females who won and lost a similar proportion of aggressive interactions were categorized as mid ranking. Females who lost more aggressive interactions than they won were classified as low ranking. This approach has several limitations, since it is based on a small sample size of decisive aggressive outcomes per female and does not account for the identity of opponents. However, several females had clear tendencies to both initiate and also win aggressive interactions or to avoid initiating and to loose aggressive interactions. As a result, this approach was effective in distinguishing clear categories of females who differed in their aggressive tendencies. Moreover the rank relationships based on this small sample size of aggressive interactions were consistent with the female dominance hierarchy as established in a prior study involving several of the same colony members [30].

To obtain a physiological index of social tension, we measured cortisol levels in urine samples collected non-invasively from focal females and their sub-group members following each focal observation. For husbandry and research purposes, all bonobos had been previously trained to urinate into plastic dishes held by zookeepers outside the fenced section of the off-exhibit enclosures [36]. Providing a urine sample was voluntary and individuals received a small food reward after urinating. Across the study, urine samples were collected at the same time of day, to insure that any differences in cortisol levels between non-reunion and reunion days could not be due to diurnal variation in cortisol release [37]. On reunion days, urine samples were collected from focal females and their sub-group members at one, 3.5 and 5 hours following reunions. Due to the delay in excretion of cortisol in urine in apes [38], the first sample should reflect baseline cortisol levels prior to reunions, the second sample should capture rising cortisol levels in response to acute stressors and the third sample should reflect decreasing levels during recovery from stressors. Females were included in cortisol analyses on days when they contributed all three urine samples.

Samples were stored at -20°C at the Frankfurt Zoo until shipment on dry ice to the Max Planck Institute for Evolutionary Anthropology, Leipzig. Samples were then stored at -20°C until the time of analysis. Samples first went through hydrolysis and solid-phase-extraction in a modification of techniques used by Hauser and colleagues [39]. In brief, steroid glucuronides were hydrolysed using **ß**-glucuronidase from *Escherichia coli* (Sigma Chemicals Co., St. Louis, MO, USA). Extracts were purified via solid phase extraction (Chromabond HR-X, 30mg, Macherey-Nagel, Dueren Germany). Steroids were extracted with tert. butyl methyl ether, evaporated and reconstituted in 70% ethanol. Samples were diluted between 1:40–1:240 and run in duplicate in an Enzyme Immunoassay provided by Coralie Munro (University of California-Davis, CA, USA). Serial dilutions of a pooled urine sample and cortisol standards gave parallel displacement curves. Inter-assay cvs for high and low concentration controls were 3.9% and 8.7%. Intra-assay cvs for high and low concentration controls were 5.2% and 12.0%. To control for variation in fluid intake and output, hormone concentrations were expressed as ng per mg Creatinine (Cr). Creatinine was measured via microtiter plate analysis based on the Jaffé reaction [40].

Statistical analyses were performed in R 3.1.1 [41] using the package Lme4 [42]. We used generalized linear mixed models (GLMM) with binomial error structure to test whether females were more likely to initiate aggression, grooming or socio-sexual solicitations with sub-group members (response variables) on reunion vs. non-reunion days (test predictor). This analysis included N = 20 pairs (representing N = 10 dyads) of focal females and adult female sub-group members who were observed on multiple non-reunion and reunion days. Each pair was observed on 5.8 (± 2.0) non-reunion and 4.6 (± 1.6) reunion days. We also tested whether dyads were more likely to have a socio-sexual interaction on reunion vs. non-reunion days. We used a linear mixed model (LMM) with Gaussian error structure to compare each female’s daily urinary cortisol levels (response variable) on non-reunion (N = 66 urine samples) and reunion days (N = 81 urine samples). The test predictor was an interaction between observation day (non-reunion vs. reunion) and the time of day when the sample was collected, to determine whether daily fluctuations in cortisol levels differed between non-reunion and reunion days. We also used a LMM to test whether each female’s daily fluctuations in urinary cortisol levels (response variable) were associated with the amount of grooming or genital contacts received during the first focal observation of the day, occurring immediately following simulated fusions on reunion days. The test predictors were interactions between each social measure (grooming or genital contacts received) and the time of day of sample collection. The data set included all focal females who provided three urine samples on at least two observation days (N = 5 females). Since we found no effect of observation day (non-reunion vs. reunion) on cortisol levels (see results), we included samples from focal females on non-reunion days (N = 27 samples) and reunion days (N = 30 samples), to increase the sample size for each focal female.

For N = 28 joiner and resident pairs (representing N = 14 dyads) that were tested on 3.1 (± 1.3) reunion days, we used GLMMs with binomial error structure to test whether receiving grooming or socio-sexual solicitations from a partner on reunion days (response variables) was associated with the likelihood of directing aggression against the partner, the dominance rank category of the partner (higher/same vs. lower rank category), the number of days that the target had been separated from the partner or whether the partner was the joiner or the resident (test predictors). We also used a GLMM to test whether the likelihood of accepting a socio-sexual solicitation (response variable) was influenced by these same test predictors.

In all models, response and predictor variables were transformed when necessary to achieve approximate normality. Individuals, dyads and observation days were included as random effects. In addition, to reduce the possibility of inflated type I error rates, we included in each model random slopes of all fixed effect predictors that varied within individuals and dyads [43]. In this way, we accounted for random variance of these factors on measures of the response variables. We assessed model stability by comparing the estimates derived from a model based on the full data set with those obtained from a model with each female subject excluded one by one. We also used the ‘vif’ function in the package ‘car’ [44], to test for variance inflation and used the ‘overdisp.test’ to test for over-dispersion. We used likelihood ratio tests to compare each model to a null model excluding the test predictors and only present results of models that differed significantly from the null.

## Results

**Fig 1:**
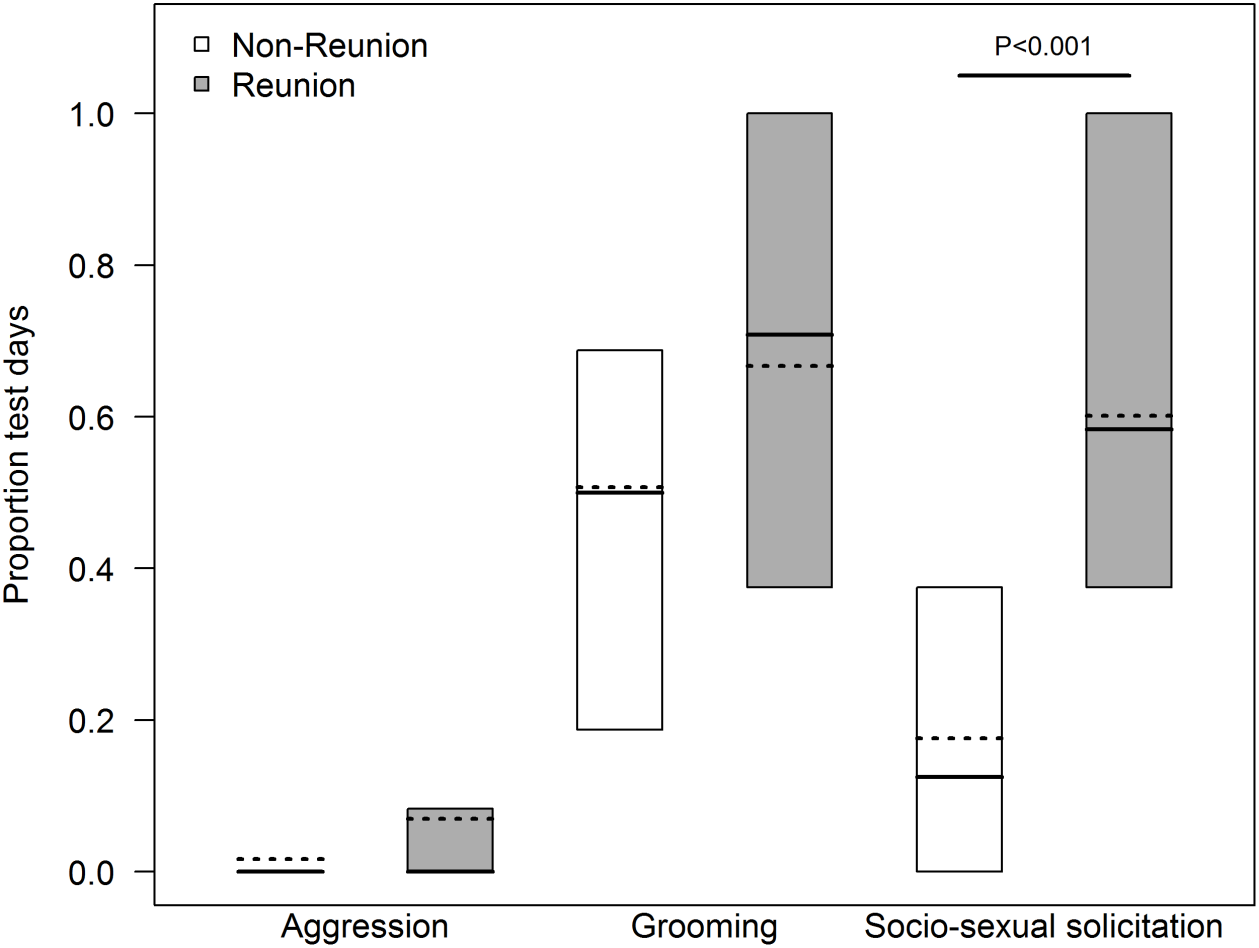
Proportion of non-reunion and reunion days during which aggression, grooming and socio-sexual solicitations occurred between pairs. Box plots indicate medians and 25–75% inter-quartile ranges. Dashed lines indicate the expected values on non-reunion and reunion days based on the corresponding model.

During 96 hours of focal observations (N = 54 non-reunion and N = 42 reunion hours), there were N = 14 aggressive interactions between focal females and sub- group members. (0.10 (± 0.22) per hour on non-reunion and 0.38 (± 0.48) per hour on reunion days). Aggression consisted of non-contact directed displays or charges. On reunion days, all aggression (N = 11 events) occurred between joiners and resident females. However, dyadic aggression did not increase between non-reunion and reunion days (likelihood ratio test, Chisq = 1.93, df = 1, P = 0.165, Fig 1). Considering all decisive aggressive interactions in which females were involved (4.33 (± 1.86) per female), females could be clearly distinguished into rank categories based on whether they won a higher, similar or lower proportion of their aggressive interactions. High ranking females (N = 2) won all or almost all (80–100%) of the aggressive interactions that they were involved in. Mid ranking females (N = 2) won between 50–67% of their aggressive interactions and low ranking females (N = 2) either never won, or won only one of their aggressive interactions.

Cortisol levels of females did not differ between non-reunion and reunion days (174.15 (± 33.69) ng/mg Cr vs. 164.82 (± 60.10) ng/mg Cr, likelihood ratio test, Chisq = 2.82, df = 2, P = 0.244, Table 1). Results were similar when comparing cortisol levels of joiners only, on reunion days vs. non-reunion days when they remained in the same sub-group (likelihood ratio test, Chisq = 2.76, df = 2, P = 0.251). Focal females and partners groomed during 20.03 hours across observation days (13.84 (± 6.89) min/hour on non-reunion and 18.22 (± 4.68) min/hour on reunion days). Within pairs, females were not more likely to groom their partner on reunion vs. non-reunion days (60.8 (± 40.8)% of reunion vs. 47.1 (± 35.2)% of non-reunion days, likelihood ratio test, Chisq = 0.951, df = 1, P = 0.330, Fig 1).

There were N = 159 socio-sexual solicitations involving focal females and other female sub-group members across observation days (0.64 (± 0.48) per hour on non-reunion and 1.41 (± 0.34) per hour on reunion days). The majority of female intra-sexual solicitations (73.0%, N = 116) occurred on reunion days, and of those, 75.9% (N = 88) occurred within the first fifteen minutes following reunions. Within pairs, females were more likely to solicit partners on reunion days (57.9 (± 33.1)% of reunion vs. 27.1 (± 21.3)% of non-reunion days, GLMM, estimate ± SE = 1.95 ± 0.54, P < 0.001, Fig 1). Of all female intra-sexual solicitations, 68% (N = 108) resulted in genital contacts or mounting and the remainder were rejected. Female socio-sexual interactions occurred 0.42 (± 0.29) times per hour on non-reunion and 1.0 (± 0.26) times per hour on reunion days. Dyads were more likely to have a socio-sexual interaction on reunion days (75 (± 42.5)% of reunion vs 44.2 (± 39.3)% of non-reunion days, GLMM, estimate ± SE = 2.63 ± 1.04, P = 0.01, Table 2). In comparison, there were only N = 16 inter-sexual solicitations between joiners and adult males across reunion days, of which 25.0% (N = 4) resulted in mounting or genital contact. Daily fluctuations in cortisol levels across non-reunion and reunion days were not associated with the amount of grooming that a female received, or with her frequency of socio-sexual interactions (likelihood ratio test, Chisq = 3.37, df = 4, P = 0.498).

**Table 2:** Results of GLMMs comparing behavior on non-reunion and reunion days (Models I-II) and identifying variables that influence socio-sexual behavior on reunion days (Models III-IV).

*Response Variable*	*Estimate*	*SE*	*P value*
I. Socio-sexual solicitation (yes/no)			
Intercept	-1.543	0.643	
**Reunion days**	**1.955**	**0.543**	**<0.001**
II. Socio-sexual interaction (yes/no)			
Intercept	-0.749	0.950	
**Reunion days**	**2.626**	**1.038**	**0.011**
III. Receiving socio-sexual solicitations (yes/no)			
Intercept	-0.969	0.715	
**Time apart (sqrt transformed)**	**1.308**	**0.453**	**0.004**
Solicitor is lower ranking	0.352	0.883	0.691
**Solicitor is resident**	**2.202**	**1.245**	**0.077**
Target aggresses solicitor	1.596	1.209	0.187
IV. Acceptance of socio-sexual solicitations (yes/no)			
Intercept	3.890	1.148	
Time apart (sqrt transformed)	-0.416	0.422	0.325
**Solicitor is lower ranking**	**-2.079**	**0.846**	**0.014**
**Solicitor is resident**	**-2.889**	**0.941**	**0.002**
Target aggresses solicitor	1.486	1.565	0.342

Significant predictors are indicated in bold.

**Fig 2:**
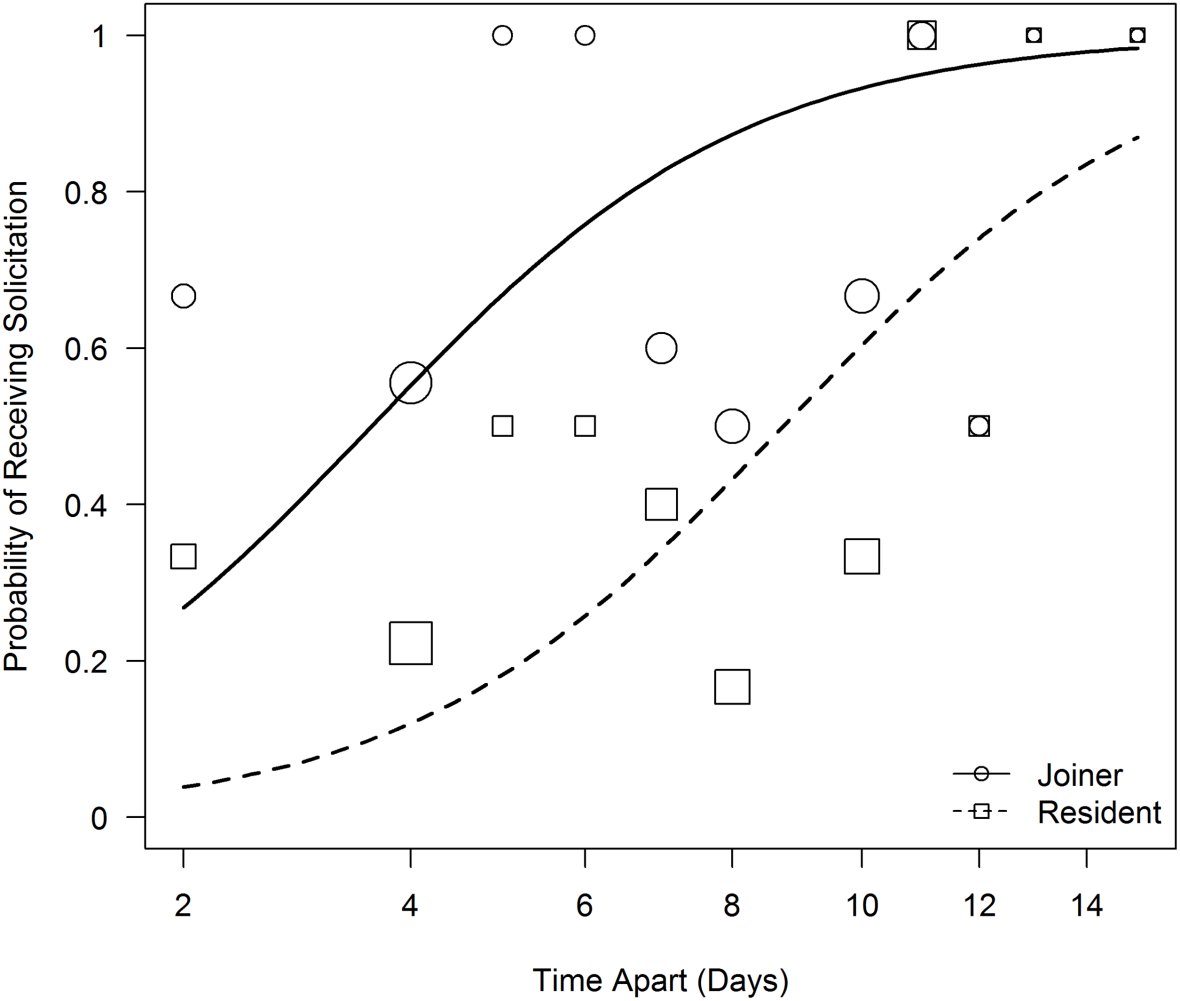
Results of a GLMM modeling the probability of receiving socio-sexual solicitations on reunion days. The x-axis is shown on the square root scale. Larger symbols indicate more pairs with the same proportional score. Regression lines indicate expected probabilities of receiving solicitations based on the model. Females were more likely to receive solicitations when they had been separated from the solicitor for longer (P = 0.004). In addition, there was a tendency for joiners to receive more solicitations than residents (P = 0.08). For full model results refer to Table 2.

On reunion days, patterns of grooming were not explained by any of the test predictors (likelihood ratio test, Chisq = 7.34, df = 4, P = 0.12), but patterns of socio-sexual behavior were influenced by the test predictors. Females were more likely to be solicited by partners when they had been separated for longer (GLMM, estimate ± SE = 1.31 ± 0.45, P = 0.004, Fig 2). In addition, joiners tended to receive more solicitations than residents (GLMM, estimate ± SE = 2.20 ± 1.25, P = 0.08, Fig 2). Females were equally likely to be solicited by higher and lower ranking partners and there was no relationship between receiving solicitations from partners and directing aggression towards partners (Table 2).

**Fig 3:**
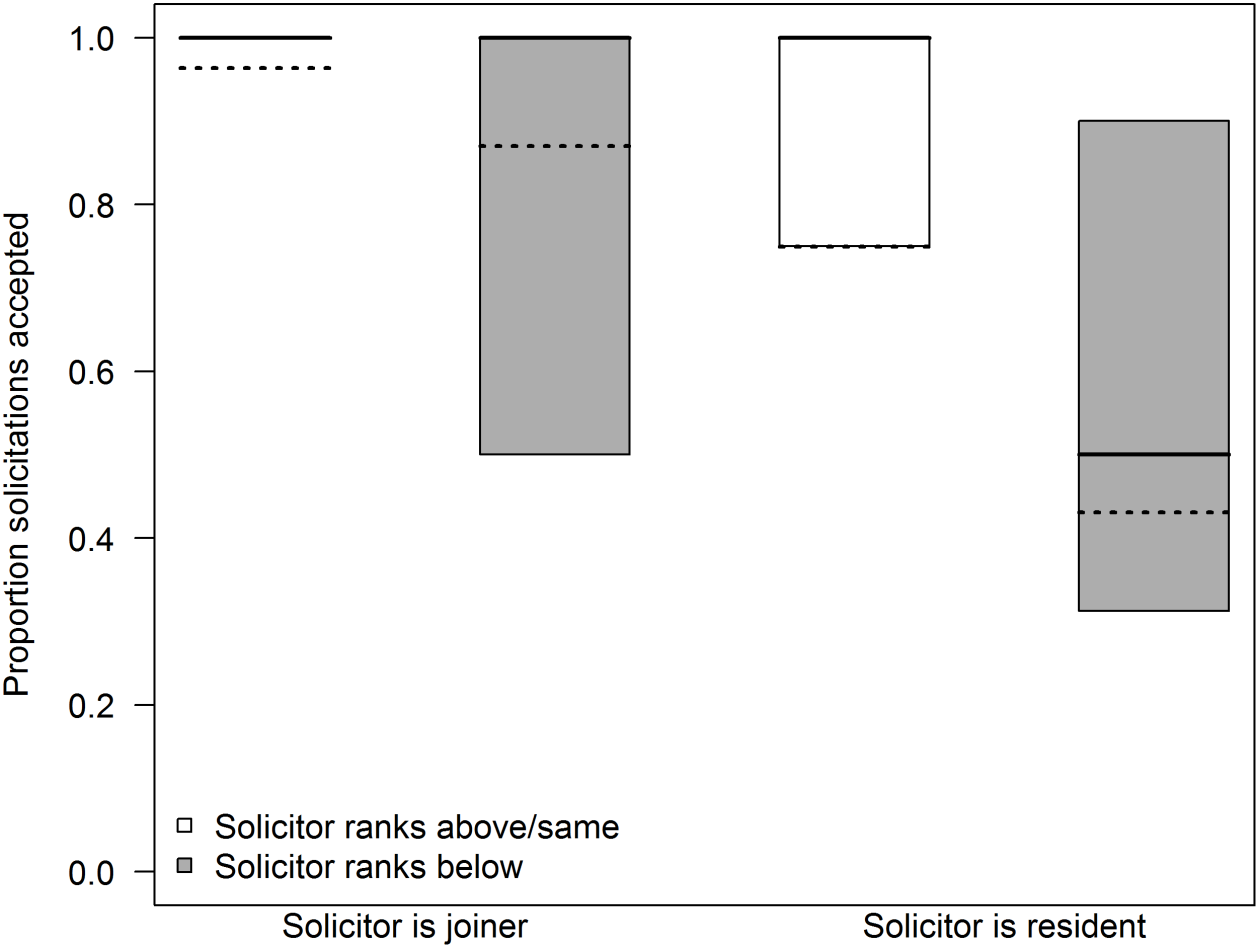
Results of a GLMM modeling the proportion of solicitations on reunion days that led to socio-sexual interactions. Solicitations by joiners were almost always accepted, while solicitations by residents were more likely to be rejected (P = 0.002). In addition, solicitations by higher-ranking females were more likely to be accepted (P = 0.014). Box plots as in Fig 1. Dashed lines indicate the expected values based on the corresponding model.

On reunion days, 68.1% of female intra-sexual solicitations were followed by genital contacts or mounting, while the rest were rejected. Residents rejected only 12.2% (N = 5) of solicitations that they received from joiners, while joiners rejected 42.7% (N = 32) of solicitations from residents (GLMM, estimate ± SE = -2.89 ± 0.94, P < 0.01, Fig 3). Joiners and residents were also more likely to reject solicitations from lower-ranking females (GLMM, estimate ± SE = -2.08 ± 0.85, P = 0.01, Fig 3). Acceptance of solicitations was not influenced by the amount of time that the pair had been separated, or by the occurrence of aggression between the pair (Table 2).

## Discussion

In species with a high degree of fission-fusion social dynamics, the flexibility to fission into smaller sub-groups or to travel independently from others is an important strategy to reduce competition over limited resources [45]. While increases in group size through fusions may provide several benefits, including enhanced protection from predation [4] and increased opportunities for exchange of social information [46], fusions are also expected to trigger increased competition and social tension, especially among reunited individuals [15], [16], [47]. Accordingly, in several species with frequent fluctuations in the size, composition and spatial cohesion of sub-groups, ritualized social interactions are common during fusions, including embraces among spider monkeys [22] and chimpanzees [24] and pseudo-penile displays among female hyenas [17], [48]. These behaviors may have varied functions including reinforcing or strengthening cooperative social bonds among allies who have been apart (chimpanzees: [24], hyenas: [48]), and also reducing the likelihood of receiving aggression among less preferred social partners (hyenas: [17], spider monkeys: [15]). In chimpanzees and hyenas, post-fusion social interactions are more frequently initiated by subordinate individuals and often involve rank-specific roles that may help to avoid or reduce conflicts by signaling the acceptance of differences in social status [17], [24].

We found little support for an increase in social tension among captive female bonobos reunited within sub-groups of varied composition and after varying periods of separation. Although all examples of aggression on reunion days occurred among reunited females, aggression remained infrequent across non-reunion and reunion days, and consisted of relatively low-intensity charges or displays that never escalated to contact aggression. Moreover, joiners and residents did not exhibit physiological indicators of stress on reunion days. It is possible that simulated fusions in captivity do not elicit strong behavioral or endocrine responses, either because the fission is never complete due to spatial limitations, or because the potential sources of heightened social tension, such as increased competition over resources, are not present. However, there is evidence that female bonobos compete over preferred food resources in captive settings [30], [49], so the weak effect of fusions on aggression and cortisol is unlikely to be explained by a lack of potential for competition in captive environments.

Although bonobos maintained limited visual, auditory and olfactory contact while in separate sub-groups, these conditions provide some parallels with field settings, where bonobo sub-groups often coordinate travel and maintain vocal contact while feeding in separate locations [50]. Moreover, reunions triggered robust behavioral responses specifically among individuals who had been apart, suggesting that simulated fissions and fusions in captivity are biologically meaningful for female bonobos. Similarly, spider monkeys who were partially visually occluded while using different areas of the same enclosure exhibited increases in embraces when first coming into contact [22], comparable to behavioral responses of wild spider monkeys during fusion events [15].

The lack of social conflict during reunions may indicate a role of socio-sexual behavior in mitigating post-fusion social tension among reunited females. Socio-sexual behavior increased immediately following fusions and occurred overwhelmingly between the joiner and other resident females, although there were also adult males present in every reunion group. Joiners and residents who had been separated for longer were more likely to solicit each other for socio-sexual interactions, as predicted if increased separation time causes more uncertainty in social interactions following fusions. While there was no direct relationship between patterns of socio-sexual behavior and patterns of aggression between pairs, the ubiquity of socio-sexual behavior among joiners and residents on reunion days may have contributed more generally towards the relatively low frequency and low-intensity of aggression across reunion days. These results provide some parallels with responses of captive bonobos when given opportunities to interact with strangers [51], [52]. In these situations as well, rates of aggression remained low and bonobos preferred to engage in affiliative and socio-sexual behavior with unfamiliar individuals rather than familiar group members. Moreover, this preference was strongest when the stranger was a female and the majority of socio-sexual interactions occurred between unfamiliar female dyads.

Socio-sexual behavior plays a prominent role in social interactions between recently immigrant and more long-term resident females [28], [53], and is believed to facilitate the rapid integration of immigrant females into social groups [28]. Among more long-term residents, socio-sexual behavior has been primarily implicated in reducing social tension in feeding contexts, such as when first entering feeding trees, or when begging from a possessor of a highly preferred food [9], [25]. Here we observed an important role of socio-sexual behavior in reinforcing and maintaining social relationships among females following separations. Genital contacts and mounting are brief interactions that involve some degree of risk in exposing vulnerable body parts. These behaviors may act as efficient signals of benign intent, as has been proposed for post-fusion ritualized social interactions in other species [17]. However, patterns of post-fusion socio-sexual behavior in this study differed from the post-fusion behaviors that have been described in chimpanzees [24] and hyenas [17] in the lack of clear dominance-based asymmetries in their initiation. Reunited females were equally likely to solicit higher- as well as lower-ranking partners for socio-sexual interactions, suggesting that maintaining tolerant associations with a diverse range of partners may be critical for females to gain enhanced benefits through group membership, regardless of rank. Such benefits may include female-biased possession and sharing of preferred, monopolizeable foods [9], and female coalitionary aid in protecting offspring from male aggression [14].

In spider monkeys, males and females engage in post-fusion affiliative embraces that do not correspond with clear dominance asymmetries between partners [15], [22] and there is no post-fusion aggression among individuals who embrace [15]. However, unlike patterns observed in this study, post-fusion embraces among spider monkeys in captive and field conditions involve only a sub-set of potential dyads who have been apart, which may explain why fusions are associated with increased rates of overall aggression in spider monkeys [15]. It would be interesting to determine whether socio-sexual behavior is as widespread among female bonobos during fusions in the wild as we found in this captive study. It would also be interesting to compare how partner choice for social interactions at fusions in bonobos and spider monkeys influences post-fusion behavioral coordination and cooperation among females.

Post-fusion initiation of genito-genital contacts among these captive female bonobos also differed from patterns of post-fusion social interactions in other species in the high proportion of solicitations that were rejected. On reunion days, joiners tended to receive more solicitations from each resident female than they initiated, indicating high motivation by resident females to engage in socio-sexual interactions with the joiner. However joiners were more selective in their acceptance of socio-sexual solicitations than were residents. In addition, higher-ranking joiners and residents were more likely to reject solicitations from lower ranking females. When a female’s solicitations were rejected, solicitors often persisted until they were successful. As a result, across reunion days 80% (N = 28) of all possible joiner-resident dyads had at least one socio-sexual interaction. By rejecting a proportion of solicitations, joiners and higher-ranking females received more solicitations for each socio-sexual interaction that they took part in and were able to have more control over the context in which their socio-sexual interactions occurred.

Rejections of socio-sexual solicitations by bonobos occur in other captive and field settings [49], although their causes have not been investigated. Rejections of solicitations may incur costs to the solicitor, both through increased time and effort required to renew solicitation attempts and possibly through decreases in social status. Conversely, having more control over the conditions under which socio-sexual interactions occur may be a means for higher-ranking females to reinforce their social status, as has been noted in other studies [25]. By becoming more desired partners for socio-sexual interactions, joiners also appeared to gain more control over their socio-sexual interactions, which may be a way for lower-ranking joiners to enhance their social standing.

In addition to the established role of female socio-sexual behavior in the gradual acceptance of immigrant females into social groups, this study suggests that socio-sexual behavior continues to play a role in helping to reintegrate resident females into social groups following fusions. Additional studies at other captive facilities designed to simulate fission-fusion social dynamics, along with improved methods for observing fusions in field settings, are critical for determining whether the patterns of post-fusion socio-sexual behavior observed here are widespread across different bonobo groups. A combination of captive and field studies would also be useful for investigating whether variation in behavioral and physiological responses to fusions may reflect variation in the quality of social relationships and correspond with differences in the extent of behavioral coordination and cooperation achieved among females following fusion events.
